# Cross-cultural adaptation of Chinese students in the United States: Acculturation strategies, sociocultural, psychological, and academic adaptation

**DOI:** 10.3389/fpsyg.2022.924561

**Published:** 2023-01-04

**Authors:** Hongling Lai, Dianjian Wang, Xiancai Ou

**Affiliations:** ^1^School of Foreign Languages, Lanzhou Jiaotong University, Lanzhou, Gansu, China; ^2^School of Education Science, Guangxi University for Nationalities, Nanning, Guangxi Zhuang Autonomous Region, China

**Keywords:** Chinese students, acculturation strategy, academic adaptation, sociocultural adaptation, psychological adaptation

## Abstract

This study was conducted with the primary purpose to gain a comprehensive understanding of Chinese students’ acculturation by examining the effects of their acculturation strategies, sociocultural, psychological adaptation on the academic adaptation. The total number of 315 international Chinese students studying in the United States participated in an online survey. The mid-point split method was used to classify the four acculturation strategies among Chinese students. The results of a Chi-square test, ANOVA analyses and hierarchical regressions reveal that separation was the most preferred acculturation strategy by the Chinese students while marginalization was the least desirable. Chinese students did the best in sociocultural adaptation but the worst in academic adaptation. However, students who achieved a good sociocultural adaptation encountered unexpected problems with their psychological adaptation. Findings also indicate that international Chinese students’ preference for separation and marginalization was associated with a better psychological and academic adaptation while integration and assimilation strategy was associated with a better sociocultural adaptation. Their academic adaptation can be predicted significantly by their psychological adaptation, not by their sociocultural adaptation.

## Introduction

Difficulties in adapting to a new environment can interfere with international students’ academic success and overall well-being. Aside from the social cultural problems experienced by many immigrants, international students can also suffer from academic difficulties due to their new educational environments (Geeraert et al., 2019; Cruwys et al., 2020; Kumi-Yeboah et al., 2020; Choy et al., 2021). International students’ acculturation is often studied in the framework of immigrants’ acculturation behaviors. So, salient variables in the acculturation models have been examined, failing to gain a global picture of how international students’ acculturation was achieved as a whole. Among the three types of adaptations, namely, sociocultural, psychological and academic adaptation the international students have been acknowledged widely to encounter, which is working predominantly and how are they interrelated? If academic adaptation is the primary concern of international students’ education, then can it be predicted by their acculturation strategy preference, or their sociocultural adaptation or psychological adaptation? A holistic understanding of the underlying mechanism in international students’ acculturation will undoubtedly help the educationists or practitioners in better understanding and working with the international students.

Many previous studies have used acculturation strategies to predict sociocultural and psychological adaptation or other outcome variables (Cao et al., 2017; Choy et al., 2021; Cohen-Louck and Shechory-Bitton, 2021; Yang et al., 2022). However, few studies on how acculturation strategies predict international students’ academic adaptation could be found. Furthermore, targeted at international students, most studies took the Western students as samples (Chirkov et al., 2008; Grigoryev and Berry, 2017; Kodja and Ryabichenko, 2019; Yu et al., 2019). Research on Chinese students’ acculturation strategies in the United States can only be seen sporadically in the literature. Even when they were studied, international Chinese students from Chinese mainland were often grouped into the category of Asian students or mixed with the students from Taiwan and Hong Kong (Wang, 2009; Zhang-Wu, 2018). Taking Hong Kong, a China’s Special Administrative Region and a former British colony, as an example, while sharing a Confucian heritage with China, the values, languages and education systems used in Hong Kong reflect a mix of Chinese and Western influences, providing a distinctive hybrid higher educational environment.

Therefore, this study intends to examine what are the most commonly adopted acculturation strategies by Chinese students as they adapt to studying in the United States. Meanwhile, the potential differential impacts of Chinese students’ acculturation strategy preferences, sociocultural and psychological adaptation on their academic adaptation are also investigated to find out any characteristics that Chinese students may demonstrate in their cross-cultural adaptation as international students in the United States. One thing is of note that the term *Chinese students* in the present study is used to refer to those from Chinese mainland who were mostly born after 1990s. The cohort of these Chinese students is different from those before the 1990s in the previous studies for several reasons. First, most of them are a generation of singletons due to China’s one child policy. These students were not as placid as the earlier, older generation or as shy in dealing with Americans since they were brought up studying in an open and multimedia learning environment.

This study supplements to the existing literature in the following two ways. First, it examines participants’ preferred acculturation strategies to gain a better understanding of the Chinese students studying now in the United States. Second, it explores how acculturation strategies, sociocultural and psychological adaptation are associated with the academic adaptation. Overall, the findings reported in this article offer guidance and implications to both academics and practitioners in better understanding and working with the international students in the cross-cultural context.

## Literature review

### International students’ academic adaptation

Previous studies on the acculturation of international students have tended to be conducted within the framework of immigrants’ acculturation. Ward and Kennedy (1994) distinguished two dimensions of acculturation: sociocultural and psychological adaptation. Sociocultural adaptation is related to the ability of adjustment or adaptation in the new culture while psychological adaptation is the sense of happiness and satisfaction (Searle and Ward, 1990). As more attention was paid to the international students, the current acculturation models of immigrants have been applied to study international students (Smith and Khawaja, 2011). Several systemic literature reviews summarized the different focuses on sources of stress, individual differences and wellbeing experienced by international students (Alharbi and Smith, 2018), as well as the predictors of psycho social adjustment of international undergraduate and graduate students in the United States (Zhang and Goodson, 2011; Choy et al., 2021).

However, considering only psychological and sociocultural adaptations in the study of international students’ acculturation is not enough. As Gilbert (2000) argued, academic culture shock is a subset of the wide range of culture shock experienced by international students and is associated with differences in the academic learning environment between international students’ home country and the host country. In the context of an upsurge of international students, another dimension of acculturation has also been proposed: academic adaptation, the process of adjusting to the new educational systems was among the major concerns for international students (Church, 1982). In particular, the differences in the academic learning environment include variations in the education system, lecture styles, classroom culture, instructional methods, teaching styles, communication with schoolmates and assessment system among countries (Lou, 2021). Adjustment to the campus environment involves adjustment to courses, adjustment with lecturers, employees and adjustment to the campus organization (Li and Peng, 2019) since international students come from different cultural, language and religious backgrounds, some of which are very different from the host culture (Alharbi and Smith, 2018).

It is of importance to pay close attention to differences in cultural values, especially in cross-cultural studies (Kristiana et al., 2022). There is a clear gap in cultural values between developed Western countries and less developed Eastern countries. While America and Europe can be considered as individualism-oriented cultures, Asia, Africa, and Latin America have strong collective values. Guided by Confucianism, Chinese culture is based on high collectivism and harmonious interpersonal relationships while the United States is a country with low power distance, typical individualism and short-term orientation (Hofstede, 2001). American culture exhibits more characteristics (e.g., autonomy, uniqueness, direct communication) which emphasize individualism, while Chinese culture reveals more characteristics (e.g., compliance, harmony) that belong to collectivism (Hofstede, 2001). Thus, set in the United States educational system, Chinese students experienced challenges learning to adapt and integrate into new educational environments (Kumi-Yeboah et al., 2020). The challenges facing Chinese students in adapting to a Western learning setting involve the preference and development of acculturation strategies that impact on their identity, as Wang and Hannes (2014) have found that the international students’ attitudes toward their home and host cultures are directly related with their academic learning experience.

### International students’ acculturation strategies

Berry (1997) conceptualized acculturation as a bi-dimensional process in which two orthogonal acculturation orientations or attitudes related to the maintenance of an individual’s native culture and the acceptance of the local mainstream culture combine to produce a range of possible acculturation strategies: (a) integration (high identification with both the heritage and the host culture); (b) assimilation (high identification with the host culture and low identification with the heritage culture); (c) separation (high identification with the heritage culture and low identification with the host culture); and (d) marginalization (low identification with both the heritage and the host culture).

According to Berry (1997), maintaining the home culture is primarily related to an individual’s psychological adaptation while adopting the host culture relates to one’s sociocultural adjustment. Thus, acculturation strategies may impact one’s adaptation outcome. While some of the previous studies indicated that the integration strategy and assimilation were strongly associated with sociocultural adaptation (Grigoryev and Berry, 2017; Kodja and Ryabichenko, 2019; Neto and Neto, 2022), other studies taking Asian immigrant students as participants showed that the integration strategy was positively related to both sociocultural and psychological adaptation (Hui et al., 2015), integration and marginalization interacted to influence only one specific domain of psychological adaptation (Ng et al., 2017). Individuals integrating bicultural identities were found to tend to achieve better psychological and sociocultural adaptation (Wang, 2009; Chen, 2015).

Empirical evidences from previous studies have also shown the choice of acculturation strategy to be an influential factor to one’s adaptation outcome. For example, integration was found to be the most preferred acculturation strategy for immigrants to have positive acculturative outcomes, and marginalization was the least preferred (Grigoryev and Berry, 2017; Urzúa Ferrer et al., 2017; Kodja and Ryabichenko, 2019; Yoo, 2021; Neto and Neto, 2022). For instance, taking 1,628 multicultural family adolescents in South Korea as samples, the study of Yoo (2021) showed that life satisfaction was highest for those who adopted integration and lower for others that chose assimilation, separation, and marginalization. And the Colombian and Peruvian immigrants living in Northern Chile tended to adapt better psychologically and socially when they chose integration and separation strategy rather than assimilation and marginalization (Urzúa Ferrer et al., 2017).

What’s more, the results of the studies on acculturation strategies varied due to the differences in race, the cultural differences in sociopolitical, historical context and traditional values and behaviors between the host culture and the heritage culture. For instance, Choi et al. (2018) found there existed significant differences in acculturation patterns and academic performance in Filipino American and Korean American adolescents’ sub-types of acculturation strategy. Numerous studies have been undertaken to determine immigrants’ preferences for acculturation strategies in different countries and with different kinds of acculturating groups (Min, 2006; Nekby et al., 2007; Stuart et al., 2010; Suinn, 2010; Lu et al., 2012; Cao et al., 2017). Yet, the results are mixed. Although many studies supported that the integration strategy is more beneficial for migrants than the assimilation, the separation and the marginalization strategy, there were still discrepancies under various cultural contexts and immigration groups. For example, in the European context, Nekby et al. (2007) concluded that the integration strategy was the most effective for the educational success of young Swedish grown-up migrants in Sweden. However, Suinn (2010) found there was a positive relationship between separation strategy and educational success in the receiving country. Similarly, researchers found that Korean immigrants used separation strategy to build successful economic and social communities to combat the social exclusion and isolation of their host country’s culture (Min, 2006). Stuart et al. (2010) concluded that the separation strategy predicted better psychological and educational adaptation for Muslim youth in New Zealand. Even for the international groups with same nationality in different host countries, the results of the studies on the acculturation strategies are inconsistent. For instance, while Cao et al. (2017) found that integration was the most commonly adopted strategy among international Chinese students in Belgium, the study by Lu et al. (2012) showed that Chinese immigrants in Australia generally adopted separation strategy.

Furthermore, besides acculturation strategies, factors such as education level, gender, length of stay, cultural distance, English proficiency and interactions with local people were found to be good predictors of successful sociocultural adaptation (Grigoryev and Berry, 2017; Ng et al., 2017; Kodja and Ryabichenko, 2019; Lou, 2021). Alharbi and Smith’s (2018) review findings highlight major stressors and show mixed results in some areas due to the lack of homogeneous samples based on country of origin or ethnicity and sometimes context differences concerning the country or university social dynamics. For example, some studies showed that males tended to assimilate less easily and meet more adaptation problems in the process of acculturation than females (Gibson, 2001); however, the study of Ho (2006) reported that skilled women usually experienced more stress than men.

### The current study

Kim (1988) regarded the acculturation process of the individual adaptation as cognitive, attitudinal and behavioral adaptation to the new cultural system. She stated that acculturation was an individual-social communication interface. Coincidentally, Bandura had explained human agency from an interactional viewpoint. According to the social cognitive theory of Bandura (1986), the environmental (social support) and personal (self-factors and self-strategies) and behavioral factors (e.g., adaptation outcomes) interact with each other to shape human functioning. According to Berry (1997), maintaining the home culture is primarily related to an individual’s psychological adaptation while adopting the host culture relates to one’s sociocultural adjustment.

Based on the literature review above, Berry’ acculturation theory, Bandura’s social cognitive theory and Kim’s communication theory, the present study was designed to examine Chinese students’ acculturation strategies in Berry’s terms and explore how acculturation strategies, together with sociocultural and psychological adaptation, are associated with academic adaptation, we propose the following research questions:

1. What is the most commonly preferred acculturation strategy by Chinese students in the United States?
2. How are the international students’ acculturation strategies, sociocultural and psychological adaptation associated with their academic adaptation?

We hypothesized that:

*Hypothesis 1*: Integation will be the most preferred strategy among the Chinese students in the United States while marginalization will be the least desirable one.

*Hypothesis 2*: Chinese international students in the United States experienced a better sociocultural and psychological adaptation, but not an academical adaptation.

*Hypothesis 3*: Acculturation strategies, together with sociocultural and psychological adaptation will influence academic adaptation. To be specific, Integration and assimilation strategy will lead to positive academic adaptation, while separation and marginalization are associated with a negative academic adaptation. Besides, both sociocultural and psychological adaptation will predict academic adaptation positively.

## Materials and methods

### Research design

This research is a cross-sectional correlation study. Due to the difficulties in collecting data from the target populations, a snowball sampling, non-probability technique was initially used.

### Participants

This research was conducted with the participation of 315 Chinese students from Chinese mainland who were studying in 19 universities in the United States. All participants must meet the following criteria: (1).

Chinese students studying at universities in the United States; (2) only Chinese students from Chinese mainland excluding Hongkong, Macau, and Taiwan due to different cultural features and educational systems of these regions from Chinese mainland; (3) voluntary. The participants included undergraduates and graduates, comprising 163 males and 152 females with ages ranging from 18 to 48 years (M = 24.06 years, SD = 3.397). Of these, 32.1% were undergraduates, 43.8% were graduates studying for Master degrees and 24.1% were graduates studying for doctoral degrees. The average length of the students’ stay in the United States was between 2 and 3 years (M = 2.35, SD = 1.16), with 94 (29.8%) staying for less than 1 year, 98 (31.1%) staying for 1–2 years, 40 (12.7%) staying for 2–3 years, and 83 (26.3%) staying for more than 3 years. As for the geographic location of the students’ universities, 68 (21.6%) participants were from universities in the east area of the United States, 72 (22.9%) were from universities in the west, 106 (33.7%) from the south, 8 (2.5%) from the north, and 61(19.4%) from Iowa State University in the central part of the United States. In terms of their majors, 46 (14.6%) of the participants were majoring in liberal arts, 171 (54.3%) in science and engineering, 75 (23.8%) in business and 23(7.3%) in other fields.

### Measures

#### Academic adaptation

The Academic Adaptation Scale (AAS) was formulated from a survey carried out by Kovtun (2010). The original survey was designed to investigate international students’ adaptation to an American college, consisting of five parts [(a) *General Academic Skills*; (b) *Psychosocial Development*; (c) *Understanding of and Comfort with Diverse Individuals*; (d) *Time Management*; and (e) *Motivation.*]. All items are measured using a 5-point Likert format (1 = extreme difficulty to 5 = no difficulty). Higher scores represented lower level of academic difficulty and higher level of better academic adaptation. The current study used the first part of the original survey relating to academic skills (Items 1 to 6, e.g., “*Write clearly and effectively in English*”). The reason for using academic skills to measure academic adaptation is that academic skills may be regarded as one of the most important factors to help international students understand their new learning system and communicate with local students and faculty members (Zhang and Goodson, 2011). The original AAS demonstrated high internal reliability (α = 0.70). In this study, both AAS Cronbach’s alpha (α) and McDonald’s ω were 0.84 (see Table 1 for the detailed analysis).

**Table 1:** Reliability.

Scale	α	Composite reliability (CR)	McDonald’s ω	Loading
Sociocultural adaptation	0.94	0.94	0.94	0.37–0.79
Psychological adaptation	0.92	0.92	0.92	0.31–0.79
Academic adaptation	0.84	0.84	0.84	0.35–0.72
Maintenance of Chinese culture	0.84	0.84	0.84	0.42–0.79
Acceptance of American culture	0.82	0.83	0.82	0.43–0.67

#### Sociocultural adaptation

The Social Difficulty Scale (SDS; Wang, 2009) was used to measure the Chinese international’ sociocultural adaptation difficulties in the United States (e.g.*, “Understanding American jokes and humor”; “Making friends with Americans”; “Finding food that you enjoy”*). The SDS was modified according to the *Sociocultural adaptation scale* by Ward and Kennedy (1999). The modified SDS in this study consisted of 22 items using a 5-point Likert format (from 1 = extreme difficulty to 5 = no difficulty; Wang, 2009, p.168). Higher scores indicated lower levels of social difficulty and higher level of better sociocultural adaptation. Previous studies have reported high internal reliability of this scale ranging from 0.84 to 0.91 (Ward and Kennedy, 1999). In the current study, both of the Cronbach’s alpha (α) and the McDonald’s ω coefficient were 0.94 for SCAS scores (see Table 1).

#### Psychological adaptation

The Psychological Adjustment Scale (PAS) by Wang (2009) was used to measure Chinese international students’ psychological adaptation. The original version of the PAS consisted of 20 items using a 5-point Likert format ranging from 1 = strongly disagree to 5 = strongly agree (e.g., *“I feel I am able to do my schoolwork as well as most other international students,” “I feel more discouraged about my future than I used to be since I came to the U.S”*). Higher scores represented higher levels of better psychological adjustment. Items using negative words were reverse coded. The Cronbach’s alpha showed high internal reliability (α = 0.92). In the current study, the scale also yielded a high Cronbach’s alpha coefficient (α = 0.92) and McDonald’s coefficient (ω = 0.92; see Table 1).

#### Acculturation strategies

The Acculturation Index (AI) by Wang (2009) was adopted to measure the Chinese international students’ acculturation strategies in this study. To adapt to the Chinese students from Chinese mainland and Taiwan, the AI was modified based on the Acculturation Index by Ward and Kennedy (1999). The original version of AI consisted of 21 cognitive and behavioral items (e.g.*, food, recreational activities, language, world view, social customs*) to measure acculturation strategies on two dimensions of the acculturation model proposed by Berry (1997): maintenance to the heritage culture and acceptance to the host culture. In order to avoid confusion and inadequacy, some of the items such as *political ideology and worldview* were canceled, the modified version of the AI consisted of 17 items with two statements under each item with the first statement related to the maintenance of Chinese culture, while the second statement represented acceptance of American culture (e.g., “*Friendship network: I maintain good friendly networks with people from my own culture; I have many American friends or friends from different cultural backgrounds*”). Participants rated each statement on a 5-point Likert scale ranging from 1 = strongly disagree to 5 = strongly agree. This led to two independent scores, which were used to measure Chinese and American cultural identification. Higher scores on each scale indicating higher identification with Chinese and American culture. In accordance with Berry’s four mode acculturation strategies, the four acculturation types were achieved through the mid-point split method suggested by Doná and Berry (1994). The AI and mid-point split method have been validated in previous studies using Chinese students as samples (Wang, 2009; α = 0.81 for cultural maintenance, α = 0.73 for participation in the host society). The internal reliability of the two sub scales in this study were fairly reliable (α = 0.84 and McDonald’s ω = 0.84 for maintenance of Chinese culture, and α = 0.82 and McDonald’s ω = 0.82 for acceptance of American culture; see Table 1).

### Procedure

The current questionnaire, which was written in Chinese, was completed voluntarily and anonymously online. The questionnaire was translated into Chinese from English and edited by two Chinese English professors, both of whom are fluent in English and studied in United Kingdom and the United States for more than 2 years. The electronic version of the questionnaire was designed *via* Survey Monkey, and was distributed through email, QQ and WeChat (two popular social media platforms used by Chinese people) in the following two ways:

At the initial stage, through personal contacts, we were able to seek help to forward questionnaire link to participants from the head of the Office of Educational Affairs of the Chinese Embassy in San Francisco, the Chinese Students’ Association in the University of Florida, Iowa State University and the International Friendship Centre at the University of Florida. The other method was to invite Chinese students in the United States to respond to the questionnaire *via* a network of the researchers’ friends and students—i.e., *via* “snowball sampling.”

The questionnaire took around 15 min to complete. 408 students responded to the questionnaire online, with 315 students providing valid responses.

### Data analysis

SPSS 26.0 was used to analyze the data. The mid-point split method by Doná and Berry (1994) was used to classify the four acculturation strategies among Chinese students. A Chi-square test was used to examine the distribution of the Chinese students’ acculturation strategies according to demographic variables. The ANOVA analyses and *Post-hoc* pair wise comparisons using the LSD test analyzed the significant differences in terms of their sociocultural, psychological, and academic adaptation among the acculturation strategies. And hierarchical regressions were performed to find out what might have influenced or predicted the Chinese students’ academic adaptation. The method of confirmatory factor analysis (CFA) was adopted to produce factor loading which was further used to calculate composite reliability (CR), average variance extracted (AVE), and discriminate validity (see Tables 1, 2).

**Table 2:** Validity: Correlation coefficients between latent variables and square root of AVE.

	Sociocultural adaptation	Psychological adaptation	Academic adaptation	Maintenance of Chinese culture	Acceptance of American culture
Sociocultural adaptation	0.67(0.45)				
Psychological adaptation	0.45^***^	0.62(0.38)			
Academic adaptation	0.30^***^	0.28^**^	0.60(0.36)		
Maintenance of Chinese culture	−0.27^***^	−0.21^*^	−0.31^***^	0.58(0.34)	
Acceptance of American culture	0.31^***^	0.27^***^	0.53^***^	−0.37^***^	0.56(0.31)

* *p* < 0.05,

** *p* < 0.01,

*** *p* < 0.001.

The values on the diagonal were square root values of AVE (AVE was in parentheses) for five scales. And the values in the cells of lower left triangle were the correlation coefficients between latent variables.

### Reliability and validity

Generally, CFA can be used to test the structural validity of the scale, but this is based on a clear understanding of the factor structure of the scale, and the structural equation requires a large sample. However, all five scales used in this study have not yet been reported their factor structure in the previous studies, so the internal structure composition is still unclear. In addition, the sample size of this study is not large enough, so it is not feasible to use CFA to test the structural validity of each scale. Therefore, we used CFA only to yield the factor loading which can be used to calculate CR, AVE and discriminate validity. In this study, to achieve the factor loading, we temporarily conducted CFA with a uni-dimensional model for each scale. The software used by CFA is Mplus 8.0.

As Table 1 shown, Cronbach’s alpha (α), composite reliability (CR), and McDonald’s ω are almost the same, they are all greater than 0.8, indicating that the five scales have high reliability, so it is feasible to express or measure each variable with composite score.

AVE is a measure for the convergent validity. Generally, AVE is greater than 0.5 indicates that a measure has good convergent validity. The AVE of all scales in this study was lower than 0.5, indicating poor convergent validity (This result may be due to the crudely uni-dimensional modeling for five scales). According to Fornell and Farcker (1981), for a reasonable discriminate validity, the square root of the AVE for variables should be greater than their corresponding correlation coefficients. In Table 2, all of the square roots of AVE for five variables on the diagonal are greater than the correlation coefficients (absolute value) below them, indicating that there is a good discriminate validity among these variables.

## Results

### Chinese students’ acculturation strategies

Based on Berry’s (1997) bi-dimensional acculturation model, a categorization of the four acculturation strategy types used by Chinese students was conducted using the mid-point split method suggested by Doná and Berry (1994). The two sub-scales of the acculturation strategy scale (maintaining Chinese culture and adopting American culture) were measured using 5-point Likert scales with 3 as the midpoint. Respondents whose mean scores fell below 3 were categorized as “low” on the scale, while those whose mean scores were greater than or equal to 3 were classified as “high” on the scale. According to this method, each respondent was classified as “high” or “low” on both scales, and consequently, this generated four types of acculturation strategy groups. The results of the mid-point split analysis are presented in Table 3, which shows that separation (*N* = 170) was the most commonly adopted acculturation strategy by this group of Chinese students in the United States, followed by integration (*N* = 103), and assimilation (*N* = 24). Marginalization (*N* = 18) was the least preferred one.

**Table 3:** Acculturation strategies among Chinese students [M ± SD].

	Separation (*N* = 170)	Integration (*N* = 103)	Assimilation (*N* = 24)	Marginalization (*N* = 18)	Overall (*N* = 315)
Maintaining Chinese culture	3.74 ± 0.51	3.64 ± 0.47	2.56 ± 0.30	2.72 ± 0.25	3.56 ± 0.60
Adopting American culture	2.47 ± 0.40	3.35 ± 0.39	3.53 ± 0.44	2.56 ± 0.23	2.84 ± 0.60

A Chi-square test was used to examine the distribution of the Chinese students’ acculturation strategies according to demographic variables (see Table 4). The results indicate a significant difference between males and females regarding their acculturation strategies. Specifically, males showed a greater preference for the integration strategy than females, and more males tend to adopt the marginalization strategy while more females adopted the assimilation strategy. The results also showed differences in the acculturation strategy distribution among students with different levels of education, i.e., a larger proportion of graduates preferred the separation strategy while more undergraduates opted for the integration strategy. The results showed no difference among students with different length of stay in terms of their acculturation strategy.

**Table 4:** The Chi-square test of distribution of acculturation strategies by demographic variables [*n* (%)].

		Marginalization	Assimilation	Separation	Integration	χ^2^
Gender	Male	13(8.0)	7(4.3)	85(52.1)	58(35.6)	8.990^*^
	Female	5(3.3)	17(11.2)	85(55.9)	45(29.6)	
Education level	UG	7(6.9)	11(10.9)	40(39.6)	43(42.6)	13.986^*^
	GMD	9(6.5)	8(5.8)	83(60.1)	38(27.5)	
	GDD	2(2.6)	5(6.6)	47(61.8)	22(28.9)	
Length of stay	Less than 1 year	5(5.3)	9(9.6)	45(47.9)	35(37.2)	4.383
	1–2 years	6(6.1)	5(5.1)	57(58.2)	30(30.6)	
	2–3 years	3(7.5)	2(5.0)	22(55.0)	13(32.5)	
	More than 3 years	4(4.8)	8(9.6)	46(55.4)	25(30.1)	

* *p* < 0.05.

UG, undergraduate; GMD, Graduate studying Master degree; GDD, Graduate studying doctoral degree.

### Sociocultural, psychological, academic adaptation, and acculturation strategies

As shown in Table 5, the three cross-cultural adaptations in the total sample occurred in the order sociocultural > psychological > academic. Repeated measures analysis of variance revealed significant differences in the pairwise comparisons between sociocultural, psychological and academic adaptation. The mean scores for academic and psychological adaptation were both below the midpoint 3; in particular, the academic strategy only yielded a mean of 2.15, suggesting that these Chinese students had the most significant problems with their academic adaptation. For sociocultural adaptation, the mean score was 3.03, suggesting that the Chinese students were able to adapt themselves relatively better to American society. In terms of their psychological adaptation, an intermediate level was achieved.

**Table 5:** Comparison of adaptations by different acculturation strategies preference [M ± SD].

	Sociocultural	Psychological	Academic
① Marginalization (*N* = 18)	3.21 ± 0.58	2.56 ± 0.59	2.38 ± 0.25
② Assimilation (*N* = 24)	3.41 ± 0.57	2.22 ± 0.76	1.47 ± 0.45
③ Separation (*N* = 170)	2.87 ± 0.62	2.65 ± 0.67	2.52 ± 0.41
④ Integration (*N* = 103)	3.18 ± 0.67	2.43 ± 0.73	1.65 ± 0.41
Total (*N* = 315)	3.03 ± 0.66	2.54 ± 0.70	2.15 ± 0.60
F	8.773^***^	4.077^**^	121.911^***^
Levene	0.083	0.804	1.406
η^2^	0.078	0.038	0.540
*Post-hoc* tests (*LSD*)	② > ③^***^(t = 4.01)	② > ③^**^(t = 2.92)	① > ②^***^(t = 7.772)
			③ > ②^***^(t = 11.507)
	④ > ③^***^(t = 3.79)		① > ④^***^(t = 7.229)
			④ > ③^***^(t = 16.866)

* *p =* 0.000 < 0.001.

** *p* < 0.01, *** *p* < 0.001.*Post-hoc* tests only shows the results of group comparisons with significant differences.

Since there was homogeneity of variance among the groups, the results of the ANOVA analyses demonstrated significant differences in students’ sociocultural, psychological, and academic adaptation situations as they adopted different acculturation preferences. There was little difference between different acculturation strategy types in terms of social adaptation and psychological adaptation(η^2^ are 0.078 and 0.038 respectively, both lower than 0.2, belonging to small effect), but there is a big difference in academic adaptation(η^2^ is 0.54, greater than 0.5, belonging to large effect). *Post hoc* pair wise comparisons using the *LSD* test indicated that the students preferring integration and assimilation experienced better sociocultural adaptation but did worse in their academic adaptation. Meanwhile, the students preferring separation and marginalization reported better academic adaptation even though they were not so good in terms of their sociocultural adaptation. Those preferring separation and marginalization had fewer problems with their psychological adaptation and achieved a better academic adaptation than the students preferring integration and assimilation.

### The interrelations between demographic variables, acculturation strategies, and sociocultural, psychological, and academic adaptation

To find out what might have influenced or predicted the Chinese students’ academic adaptation, hierarchical regressions were performed (see Table 6). Prior to the regression analysis, the acculturation strategies were dummy-coded with integration as the reference category.

**Table 6:** Hierarchical regression analysis of the effects on academic adaptation.

	Model 1		Model 2		Model 3	
	β	t	β	t	β	t
Gender	−0.046	−0.826	−0.033	−0.852	−0.038	−0.986
Age	0.056	0.942	0.009	0.222	0.011	0.276
Education level	0.127^*^	2.101	0.033	0.786	0.048	1.152
Length of stay	0.057	1.005	0.038	0.968	0.041	1.061
Separation			0.717^***^	16.597	0.695^***^	15.850
Assimilation			−0.076	−1.839	−0.064	−1.558
Marginalization			0.280^***^	6.875	0.279^***^	6.918
Sociocultural					0.047	1.068
Psychological					0.089^*^	2.092
F	2.499^*^		52.447^***^		42.697^***^	
R^2^	0.031		0.545		0.558	
∆R^2^	——		0.513^***^		0.013^*^	

*p < 0.05,

*** *p* < 0.001.

Table 6 indicates that academic adaptation cannot be predicted significantly by demographic variables. After controlling for demographic variables, students adopting separation (β = 0.695, *p* < 0.001) and marginalization (β = 0.279, *p* < 0.001) experienced better academic adaptation than those who adopted integration, while no significant difference in academic adaptation was found between assimilation (β = 0.076, *p* > 0.05) and integration. Furthermore, after controlling for both demographic variables and acculturation strategies, it was observed that academic adaptation could not be predicted significantly by sociocultural adaptation (β = 0.047, *p* > 0.05), but by psychological adaptation (β = 0.089, R^2^ = 0.558, *p* < 0.05).

## Discussion

Focusing on the Chinese mainland students in the United States, this study investigated Chinese students’ acculturation strategies and their interrelations with sociocultural, psychological and academic adaptation. The results reveal that separation was the most preferred acculturation strategy by the Chinese students while marginalization was the least desirable. Chinese students did the best in sociocultural adaptation but the worst in academic adaptation. Separation and marginalization was associated with better psychological and academic adaptation while integration and assimilation was associated with better sociocultural adaptation.

The first result showed that separation was the most preferred acculturation strategy among Chinese students, followed by integration and assimilation. The findings did not confirm our Hypothesis 1 and was inconsistent with previous findings that integration is the most preferred acculturation strategy for immigrants (Cao et al., 2017; Grigoryev and Berry, 2017; Kodja and Ryabichenko, 2019; Yoo, 2021). However, the result accorded with Neto et al.’s (2005) research on Portuguese immigrants’ cross-cultural adaptation in Germany, Dey and Sitharthan’s (2017) study of acculturation among Pakistani adolescents. One of the possible reasons is that unlike the immigration samples in some of the previous studies, Chinese international students are mostly short-term visitors in a host country to finish their studies and return to their home country. According to the “2019 Report on Employment & Entrepreneurship of Chinese Returnees” [Centre for China and Globalization (CCG), 2020], more overseas Chinese students are returning to China for career development due to China’s booming economic growth and relatively unfavorable economic, political, and social environments overseas, involving aspects such as work and immigration policies. Most Chinese students do not intend to stay permanently in the host culture, intending rather to finish their higher education and then return home. Forty percent of them chose to come back to China for work because they have a positive attitude about the nation’s future economic development, and about 60% said they returned to reunite with their families [Centre for China and Globalization (CCG), 2020]. Therefore, they may be unwilling to abandon their traditional cultural norms in a host country as they do not intend to stay permanently in the host culture. Furthermore, based on the theory of Minoura (1992) of a critical period for learning culture which indicated that the sensitive period for learning a culture is from birth to 15 years old, it is hard for immigrants to modify their cultural values or even abandon them after that. This also may be observed not only in the popularity of separation among Chinese students but also in their integration. Even when they do make efforts to integrate into American culture (M = 3.35), they also have a similar desire to identify themselves primarily with their home culture (M = 3.64). Immigrant groups can maintain their cultural heritage while they also seek contact with the new host culture (Montgomery et al., 2021).

As for the result of marginalization to be the least favorable strategy, it coincides with many previous studies (Cao et al., 2017; Grigoryev and Berry, 2017; Kodja and Ryabichenko, 2019; Montgomery et al., 2021; Yoo, 2021) and it confirmed the Hypothesis 1. What is new in this study is that as with the other acculturation strategies, marginalization has little to do with length of residence, but is closely related to educational level and gender. Males are prone to adopt marginalization while females prefer assimilation, and graduates prefer separation while undergraduates prefer integration and assimilation. These facts suggest that acculturation strategy preference may be affected by one’s gender and maturity.

The second finding is that separation and marginalization was closely associated with better academic adaptation while integration and assimilation was associated with better sociocultural adaptation. This result partially accords with previous studies (Neto et al., 2005; Cao et al., 2017; Grigoryev and Berry, 2017; Kodja and Ryabichenko, 2019; Yoo, 2021) showing that both integration and assimilation strategy were possibly the best strategies in cross-cultural adaptation. However, these previous studies came to this conclusion focusing only on sociocultural and psychological adaptation; the current study extended the domain to academic adaptation and discovered the unexpected role of the separation and marginalization strategies in international students’ academic adaptation. A possible reason for this is that excessive and extended study after class as well as cultural barriers may restrict Chinese students’ social interactions with the local people and lead them to feel socially excluded from the host culture (Grigoryev and Berry, 2017), for more Chinese students studying abroad are willing to go back to China for their career development after finishing their study [Centre for China and Globalization (CCG), 2020]. Good sociocultural adaptation will not be possible for them, but spending more time and energy on their study ensures better academic adaptation. The heritage culture affected Chinese students’ academic features in class more strongly than the host academic context (Cao et al., 2021). And the local people in the receiving society were more willing to communicate with the immigrants adopting integration and assimilation strategy than those with separation strategy (Montgomery et al., 2021).

One thing that cannot be ignored is that these Chinese students in the United States did the worst in academic adaptation (M = 2.15) and the best in sociocultural adaptation (M = 3.03). This is consistent with our hypothesis 2 and this finding of better sociocultural adaptation in the United States evidences that the new generation of Chinese students are more open-minded, better at English and find it easier to communicate with others than previous generations, since they have been brought up in a globalized world. In spite of the significant difficulties and challenges they experience during their acculturation process, they may also feel that learning and living in another culture is helpful for their personal growth, and enjoy experiencing life from different perspectives. This may explain why integration is the second most important acculturation strategy among Chinese students, predicting better sociocultural adaptation. Nevertheless, the findings that their biggest problem is still academic adaptation suggest low efficiency in their study, even though they are seen as a group characterized by diligence and perseverance (Meng et al., 2018). More cross-cultural academic adaptation programs are needed so that guidance or coping strategies can be provided to help them out.

Another phenomenon worthy of note is that Chinese students who endorse separation and marginalization also show better psychological adaptation than those preferring assimilation and integration. This can be explained by the development of technologies and the internet (Alamri, 2018; Kumi-Yeboah et al., 2020; Raja et al., 2021). Although international students are far away from their home country, social media such as WeChat and QQ provides successful and cheap links with parents and friends at their home countries, keeping attached with the news, information, politics and other home countries’ activities, which will naturally reduce their sense of loneliness and increase their life satisfaction (Raja et al., 2021). They used the social media to socially adjust in educational settings (Wang et al., 2015). Social support from family and fellow countrymen will help students to maintain cultural identities and practices, and reduce homesickness and disorientation (Ng et al., 2017; Raja et al., 2021). Besides, Chinese movies, TV programs, music, and newspapers are available online, which can help students a lot psychologically although it may not help them to assimilate into the host culture. As the primary source of Chinese students’ social support in academic learning, co-national peers help the Chinese students have a better psychological adaptation (Cao et al., 2021). What’s more, international students maintained close relationships with home-country peer circle, and had few interactions with local people outside of campus (Grigoryev and Berry, 2017).

To our surprise, those students who wished to be integrated or assimilated into the host culture had a problem not only with psychological adaptation, but also with academic adaptation. It seemed not possible for these integrated or assimilated students to do better psychologically, socially and academically (Chirkov et al., 2008), and they were likely to have more psychological or academic adjustment issues. Clearly their psychological problems cannot be explained superficially, there must be in-depth reasons underlying these issues. One of the possible explanations may be that it is hard for Chinese students to integrate into American culture due to the limited English language proficiency though they are willing to. Language barriers and communication problems are stressors for Chinese students who wished to be integrated (Xing et al., 2020). In addition, Chinese students are educated to firmly believe in modesty, face saving and emotional restraint in social contexts, which make Chinese students may encounter difficulties in integrating into American culture (Wang and Mallinckrodt, 2006). Host nationals’ lack of willingness to communicate with culturally different others also make Chinese students encounter difficulties in fitting into American society featured by (Dunne, 2009). International students who wished to be integrated or assimilated into the host culture may feel depressed when they realized that they are different from Americans and it is hard for them to develop close relationship with local Americans due to the lack of identification with American culture, which cause much psychological distress for them (Li et al., 2021).

Another finding is that Chinese students’ academic adaptation can be significantly predicted by their psychological adaptation, but not by their sociocultural adaptation, and it is strongly associated with their attitudinal choice of acculturation strategy types. This finding supports Krashen and Scarcella’s (1978) learning theory of affection in that the individual affective factor has a big role to play in students’ learning. Particularly when the sociocultural environment is difficult to change, affective factors may be decisive in terms of learning outcome.

In a summary, from a cross-cultural perspective, our findings have shown that the international students’ choice of acculturation strategies varied by ethnicity or the destination country, sociopolitical and economic context of their home country, and individual’s personality and gender. Concerning with better academic adaptation, either separation or integration strategy could be most commonly adopted by international students, which is different from the other immigration groups’ choice of acculturation strategies. Due to the special immigration motivation and features, international students’ academic adaptation could be better predicted by their psychological adaptation. Affective factors influence international students’ academic achievements more than the sociocultural factors.

## Conclusion

The present study has produced several findings. Separation was the most preferred acculturation strategy by the Chinese students in the United States while marginalization was the least desirable. Chinese students did the best in sociocultural adaptation but the worst in academic adaptation. However, those who experienced a better sociocultural adaptation were found to have unexpected problems with psychological adaptation. Separation and marginalization strategies was closely associated with a better psychological and academic adaptation while integration and assimilation strategies a better sociocultural adaptation. Students’ academic adaptation can be predicted significantly by their psychological adaptation, but not by their sociocultural adaptation.

These findings provide specific guidelines for future research as well as for practitioners. Taking the Chinese students in the United States as the participants, our study takes a significant step forward by illuminating the academic adaptation of international students from perspectives of acculturation strategies preference and the interrelationships among the different aspects of international students’ acculturation. The findings can help us to better understand international students’ acculturation processes as a whole with the underlying mechanism discovered, which in turn can enlighten the further development of acculturation theory. Though, a comparative or similar study of samples of different nationalities is expected to be conducted to see how these findings may work on other international students, for the present study has just shown the characteristics of Chinese international students’ acculturation in United States. Nevertheless, while having enriched the current literature of acculturation research theoretically, our study practically provides guidance and implications to both academics and practitioners in better understanding and working with the international students in the cross-cultural context, as the new findings of Chinese international students would be taken as reference for educationists or teachers out of their problems, guiding international students to achieve better academic performances.

## Implications and limitations

The present findings have several implications for counseling practice. Firstly, since half of the Chinese students adopted separation strategy as their most commonly-used acculturation strategy, this implied that different from the findings made in the previous studies, the counselors in the host universities need to keep an eye on the recent developments of international students’ acculturation research, which can help them to discard stereotyped perceptions of their target clients. Secondly, while providing the counseling service to international students, counselors may need a different counseling perspective which is specific to their preeminent difficulties, cultural norms and philosophy, e.g., developing targeted cross-cultural academic training programs. Providing a place for international students to develop their bicultural learning competence as an area for intervention may lessen their cross-cultural academic stress. As to the finding that students who were discovered willing to be assimilated into the host culture, are experiencing more psychological and academic difficulties, counselors may explore the solutions to their problems at the social and community level since more might be involved in oppression and racism, etc. Intercultural communication activities are suggested to be held in the university or the community to promote the understanding of different cultures in the long-term.

There are also several limitations that may be overcome in future studies. First, this study mainly used self-report scales. Future studies can use objective measures. Second, cross-sectional study was conducted in this study due to the inconvenience of collecting the data. Longitudinal studies should be conducted in future to investigate changes in acculturation strategies and the three dimensions of acculturation among international students at different points in their stay in the host culture. Third, Considering the increasing numbers of undergraduate students from Chinese mainland studying abroad, future research could be done to investigate the differences in academic adaptation among Chinese students graduating from high schools in China and those who attended high schools or community colleges in the host country. Finally, more detailed studies are also needed to explore the emotional factors that might influence international students’ preferences for different acculturation strategies, such as their personality and maturity, and social factors, e.g., the issue of racism, and acceptance by the local people.

## Data availability statement

The raw data supporting the conclusions of this article will be made available by the authors, without undue reservation.

## Ethics statement

The studies involving human participants were reviewed and approved by Lanzhou Jiaotong University Research Committee. The ethics committee waived the requirement of written informed consent for participation.

## Author contributions

HL: conception and design, drafting the article, and revising it critically for important intellectual content. DW: design, collection, and analysis of data and writing-review and editing. XO: analysis of data. All authors contributed to the article and approved the submitted version.

## Funding

This study was supported by Foundation of a Hundred Youth Talents Training Program of Lanzhou Jiaotong University and SFLEP National University Research Project (2022GS0015).

## Conflict of interest

The authors declare that the research was conducted in the absence of any commercial or financial relationships that could be construed as a potential conflict of interest.

## Publisher’s note

All claims expressed in this article are solely those of the authors and do not necessarily represent those of their affiliated organizations, or those of the publisher, the editors and the reviewers. Any product that may be evaluated in this article, or claim that may be made by its manufacturer, is not guaranteed or endorsed by the publisher.
